# New Insights into the Degradation Path of Deltamethrin

**DOI:** 10.3390/molecules26133811

**Published:** 2021-06-22

**Authors:** Federica Aiello, Marcel G. Simons, Jan W. van Velde, Paulo Dani

**Affiliations:** 1 ECG-MAS, Expert Capability Group—Measurement and Analytical Science, Nouryon B.V., 7418 Deventer, The Netherlands; marcel.simons@nouryon.com (M.G.S.); jan.vanvelde@nouryon.com (J.W.v.V.); 2 National Research Council (CNR), Institute for Chemical and Physical Processes (IPCF), 56124 Pisa, Italy

**Keywords:** NMR spectroscopy, analytics, deltamethrin, insecticidal paints

## Abstract

Pyrethroids are among the insecticidal compounds indicated by the World Health Organization for mitigation of vector-borne diseases. Active deltamethrin (with chiral configuration α-*S*,1-*R*-*cis*) is one of the most effective pyrethroids characterized by low toxicity to humans, and it is currently tested as active ingredient for insecticidal paints. Nevertheless, several degradation processes can occur and affect the insecticidal efficacy in the complex paint matrix. In the present study, a detailed NMR analysis of deltamethrin stability has been carried out under stress conditions, mimicking a water-based insecticidal paint environment. Two novel by-products, having a diastereomeric relationship, were identified and their structure was elucidated by combining NMR, HPLC, GC-MS, and ESI-MS analyses. These compounds are the result from a nucleophilic addition involving deltamethrin and one of its major degradation products, 3-phenoxybenzaldehyde. Given the known toxicity of the aldehyde, this reaction could represent a way to reduce its concentration into the matrix. On the other hand, the toxicology of these compounds towards humans should be addressed, as their presence may adversely affect the performance of deltamethrin-containing products.

## 1. Introduction

Vector-borne diseases are among the main causes of illness and death; in the past 15 years, more than 600,000 cases of such diseases have been reported only in the US [1]. Mosquitoes are the most common vector together with ticks, related to the spread of malaria, Dengue fever, Zika virus, Chikungunya, and yellow fever. The most common solutions for preventing malaria involve the use of Long-Lasting Insecticide treated Nets (LLINs), where an insecticide is incorporated into the net fabric, or of the Indoor Residual Spraying (IRS) method, which consists in spraying residual insecticide on the interior walls of houses [2]. Both solutions have a limited effect though, and the need to find more effective vector control strategies is still compelling [3]; a recent approach promotes the use of Insecticide-Treated durable Wall Linings (ITWL) for protecting interior house walls [4].

The results achieved in paint technology in recent years are promoting this field as a new efficient vector control strategy. Recently, formulations commonly termed as insecticidal paints (IPs) have been developed; the active ingredient is added to the paint to provide a “ready-to-use” product in which the insecticide is released in a controlled way once the paint is dried [5,6,7].

Paints in general can be water-based (WB) or solvent-based (SB), the former being preferred for application in confined or poorly ventilated spaces, where solvent evaporation might be harmful or a nuisance. Water-based paints are generally preferred also because their use helps in reducing volatile organic compounds (VOCs) emissions, albeit temperature and humidity can constitute an issue for the stability of the formulation [8,9]. However, insecticides normally have low solubility in an aqueous environment, thus making it necessary to find an ingredient active at low concentrations (the insecticide content is usually kept between 0.1% and 4% wt for avoiding precipitation) [10].

Among WHO-recommended insecticides, pyrethroids are the most employed for insecticide-treated nets, thanks to their relatively low toxicity towards humans, their moderate cost, and the duration of their insecticidal activity [11]. Therefore, they represent good candidates as active ingredients in IPs [12,13].

**Figure 1:**
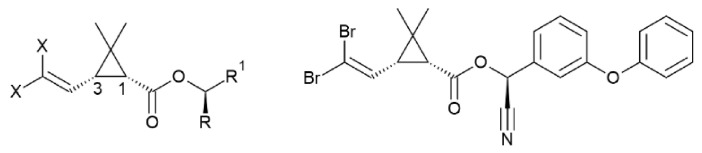
Chemical structure of (**left**) pyrethroids and (**right**) active (α-*S*,1-*R*-*cis*) deltamethrin (*a*-DLM).

Pyrethroids are synthetic insecticides known since 1970s [14], widely used in agriculture, industry, and for controlling insect pests spreading [15,16]. They are ester compounds whose basic structure consists in an aromatic alcohol and a cyclopropyl carboxylic acid (Figure 1). The stereochemistry around the aliphatic ring is usually expressed by clarifying the chiral configuration of carbon 1 and then referring to carbon 3 as *cis* or *trans* depending on the relative position of the substituents (1-*R*/1-*S*-*cis* if they are facing the same side, 1-*R*/1-*S*-*trans* in the opposite situation) [17].

One of the most frequently and widely used pyrethroids is deltamethrin, often employed for the control of household insect pests [18,19,20,21]. The presence of three chiral centers (Figure 1) translates into eight possible different stereoisomers, with only one of them having insecticidal activity. Active deltamethrin (*a*-DLM), depicted in Figure 1, has an *S* configuration at the α−benzyl carbon and a 1-*R*-*cis* configuration at the cyclopropane ring [22,23].

Degradation of deltamethrin can follow different paths, depending on the experimental conditions. UV-irradiation has been observed to cause ester cleavage, photooxidation, photoisomerization, decyanation, and dehalogenation [24,25]. In soil, degradation is mainly hydrolytic, photolytic, and microbial promoted [26]. Despite its low water solubility (<0.2 μg/L at 25 °C) [27], DLM degrades in an aquatic environment especially by oxidation and ester hydrolysis [28]. Table 1 gives an overview of the main degradation products [13,29,30,31,32].

The insecticidal activity of DLM can be disrupted not only by chemical degradation but also by stereochemical modifications of its chiral centers (Figure 1). The most labile chiral center comprises the α−benzyl carbon atom which isomerizes relatively easily, giving rise to inactive deltamethrin (*i*-DLM), with chiral configuration α-*R*,1-*R*-*cis* [30].

Given the fact that DLM is a very effective insecticide with low toxicity for mammals, it constitutes a good candidate as an active ingredient for indoor applications, such as ITWL [4] and in insecticidal paints. A study published in 2004 investigated the efficiency of the insecticide embedded in a solvent-based paint (1% wt) [33], and a patent filed in the same period evaluated the synergistic effect of DLM and piperonyl butoxide, used to inhibit insect resistance [34].

Even if both SB and WB paints are indicated as suitable matrices, the most interesting formulation is the latter, since it allows overcoming the problems related to application in enclosed spaces, as previously pointed out. Nevertheless, some aspects must be considered during the design of a water-based paint formulation containing deltamethrin as an active ingredient: its low water solubility, sensitivity to alkaline environment (WB paints usually have a pH between 8 and 9) [10], and to other degradation paths possibly emerging from such complex matrixes.

Having precise information on the degradation products of deltamethrin in a WB paint-like matrix is then necessary for a clear understanding of the pathways that can take place and the related mechanisms. Ultimately, such understanding could contribute to more stable and environmentally friendly IP formulations, using lower concentrations of insecticide with the same or improved efficacy.

This study intends to investigate the stability of *a*-DLM by identifying its degradation products in a solvent system mimicking a water-based paint (i.e., slightly alkaline and containing about 20% of water). Nuclear Magnetic Resonance (NMR) spectroscopy was selected as the main analytical technique for carrying out the study, as no sample pre-treatment is needed for the analysis, and information on the molecular alterations caused by degradation processes can be easily obtained [35,36]. NMR spectroscopy allowed identifying and quantifying the different by-products developed overtime and elucidating the degradation mechanisms related to their formation. Besides the by-products already known, a new pair of diastereomeric compounds were isolated and identified; the analysis of the degradation mechanism allowed categorizing them as secondary by-products of deltamethrin hydrolysis.

## 2. Results and Discussion

### 2.1. Analysis of Active and Inactive Deltamethrin

To mimic the solvent composition used in water-based IPs, deuterated acetonitrile (ACN-d_3_) was selected as co-solvent. In this medium, *a*-DLM converts to its inactive stereoisomer *i*-DLM quite slowly compared to other water miscible organic solvents. The ACN/H_2_O weight ratio was kept at 80:20, based on a patent filed in 2014 where water is added to a paint formulation up to 23% [37].

**Figure 2:**
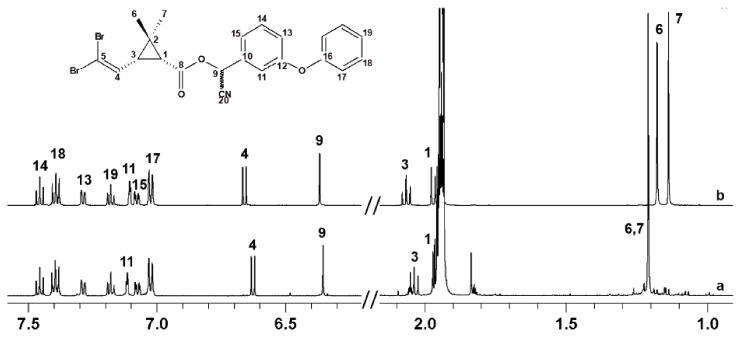
^1^H NMR (600 MHz, ACN-d_3_/H_2_O 80:20 m/m, 300 K) spectra of (**a**) *i*-DLM and (**b**) *a*-DLM.

Figure 2b and Figure S1 show the proton (^1^H) spectrum of *a*-DLM recorded in the selected solvent mixture, with aromatic protons resonating between 7.50 and 7.00 ppm; the doublet signal belonging to the olefinic proton H4 and the singlet belonging to α-proton (H9) are centered between 6.70 and 6.30 ppm. Methyl and cyclopropyl ring protons lie between 2.10 and 1.00 ppm, with solvent signal (1.94 ppm) partially overlapping proton H1 doublet.

Ideally, analysis of a compound stability via (solution) NMR spectroscopy requires all formed species to have at least one overlap-free resonance, to enable their unambiguous detection and quantitation. In case of deltamethrin, the NMR profile of Table 1 by-products was known and therefore they were expected to be distinguishable from each other and from DLM in the solvent system utilized in this investigation. It was also anticipated that the inactive diastereomer, generated from *a*-DLM by C9 epimerization, would have some differences in chemical shift with respect to the active form. As Figure 2 shows, the resonances of methyl protons 6 and 7, which clearly differ in *a*-DLM, coalesce at a higher chemical shift in *i*-DLM. Protons H3 and H1 are instead slightly shifted at lower chemical shifts in the inactive species, as well as the alkenyl proton H4 and the benzyl proton H9 (see Supplementary Materials for isolation of *i*-DLM). The calculated chemical shift variations are reported in Table S1.

### 2.2. Stability Studies and Identification of By-Products

Once confirmed that in the chosen experimental conditions it is possible to discriminate between the active and inactive form of the insecticide, the NMR analysis focused on monitoring *a*-DLM stability overtime. Emphasis was given to degradation rate and by-products formation in dependence of the temperature and the alkalinity of the water used for preparing the solvent mixture. Two different solutions of *a*-DLM were prepared in ACN-d_3_/H_2_O (80:20 m/m) using alkaline water at pH 8 (solution A) and 9 (solution B), respectively; one portion of each solution was used for monitoring the degradation at room temperature whereas the rest was kept under stirring at 40 °C. Stability of deltamethrin in the four cases was monitored over a four-week period.

**Figure 3:**
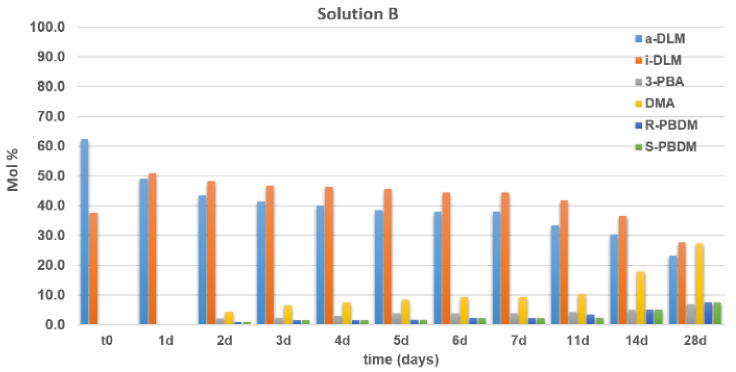
Degradation of *a*-DLM over time at room temperature in solution B. DLM: deltamethrin; 3-PBA: 3-phenoxybenzaldehyde; DMA: *cis*-decamethrinic acid; PBDM: α-3-phenoxybenzoyl-deltamethrin.

Water alkalinity clearly influences the degradation rate of *a*-DLM, as shown in Figure S3. The comparison between the spectra suggests that *i*-DLM is present in relatively low amount (Table S2) in solution A. On the other hand, in solution B, the degradation is significantly faster, and 1/3 of the starting *a*-DLM is already converted into its inactive form a few minutes after the sample preparation (Figure 3 and Table S3).

**Figure 4:**
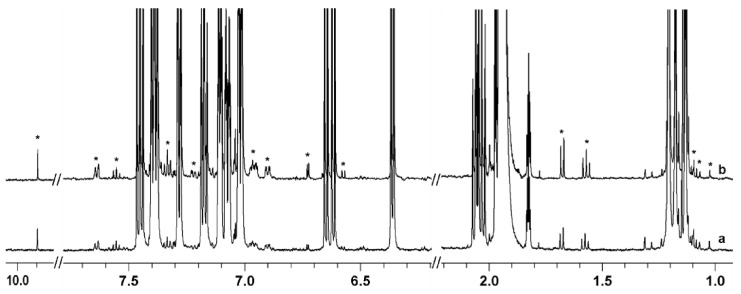
^1^H NMR (600 MHz, 300 K) spectra of *a*-DLM (9.2 mM) recorded after 48 h from preparation and kept at room temperature; (**a**) solution A and (**b**) solution B. (*) indicates signals belonging to new species.

The interconversion *a*-DLM/*i*-DLM rapidly advances within the first 24 h in both solutions, when it reaches an equilibrium (Figure 3 and Figure S4). After two days from sample preparation, it is possible to notice the appearance of new signals belonging to other species (Figure 4), present in both samples but with higher concentration in solution B. The corresponding by-products keep increasing their concentration over the analyzed period, at the expenses of both active and inactive deltamethrin.

The chemical shift of these new signals can already give some hints on the nature of the corresponding compounds, such as the singlet resonance centered at 9.90 ppm, which is indicative of an aldehyde. New signals are found in the aromatic region, as well as in the benzylic and double bond spectral region (6.80–6.50 ppm), in the methyl region and between 1.70 and 1.50 ppm (Figure 4).

**Scheme 1:**
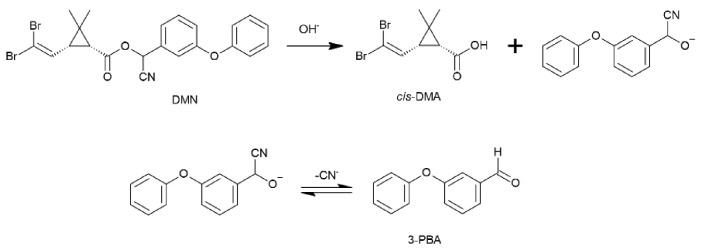
Basic hydrolysis of DLM with formation of DMA and 3-PBA.

Taking into consideration the presence of an ester functionality in deltamethrin, it is reasonable to expect, in the chosen experimental conditions, the occurrence of a basic hydrolysis reaction with consequent formation of the corresponding 1-*R*,*cis*-decamethrinic acid (DMA) and 3-phenoxybenzaldehyde cyanohydrin, both known by-products (Table 1). The latter converts into the corresponding 3-phenoxybenzaldehyde (3-BPA) by elimination of cyanide (Scheme 1); even though this is a reaction usually shifted towards the formation of the cyanohydrin, here the equilibrium lies to the formation of the aldehyde, driven by the loss of cyanide.

These results indicate that, in both solutions A and B, the main reaction taking place in the first 24 h is the epimerization of *a*-DLM α carbon, originating the inactive diastereomer *i*-DLM. This phenomenon is then followed by a sluggish chemical degradation driven by basic hydrolysis. It is worth noting that both chiral and chemical degradation rates are dependent from the water pH and temperature, being faster under stressed conditions (see Tables S4 and S5). However, neither the pH nor the temperature influences the chemical nature of the products formed. Moreover, the stereochemical degradation has been confined to involve only the DLM α carbon; the other two chiral centers remained unaltered.

DMA and 3-PBA identities were confirmed by comparison with the NMR spectra of the pure compounds and by GC-MS analysis of the stressed sample (see Supplementary Materials). Interestingly, in the analyzed samples, the amount of DMA present in solution is always higher than 3-PBA, suggesting that the aldehyde is involved in additional reactions. Not all the new resonances present in the proton spectra of the stressed samples could be assigned to DMA or 3-PBA, so the presence of additional by-products was hypothesized. To identify these components, the mixture was submitted to preparative liquid chromatography and the isolated fractions were analyzed via NMR.

### 2.3. Identification of Unknown By-Products

**Figure 5:**
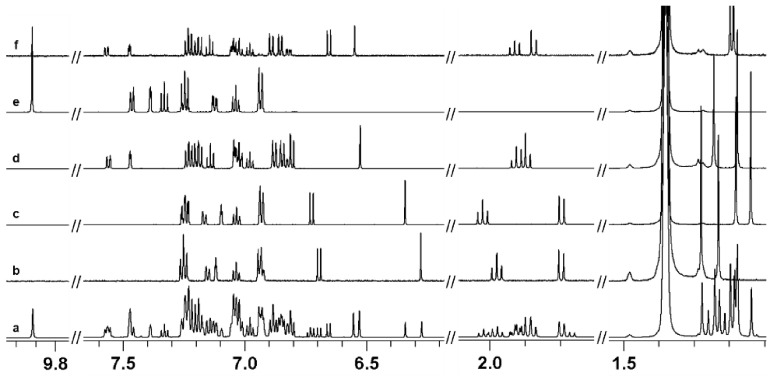
^1^H NMR (600 MHz, C_6_D_12_, 300 K) spectra of (**a**) stressed sample and compounds isolated from fraction collection: (**b**) *i*-DLM, (**c**) *a*-DLM, (**d**) *S*/*R*-PBDM, (**e**) 3-PBA, and (**f**) *R*/*S*-PBDM.

The chromatographic fractionation was performed in *n*-hexane/isopropanol 95:5 *v*/*v*. Figure 5 shows the ^1^H NMR spectrum of the original sample mixture (DLM in solution B after four weeks) and of the collected fractions dried and redissolved in deuterated cyclohexane (C_6_D_12_). It is important to keep in mind that decamethrinic acid does not dissolve in such apolar medium, therefore it is not expected to be found either in the ^1^H NMR spectrum nor in the chromatogram of the analyzed mixture.

**Figure 6:**
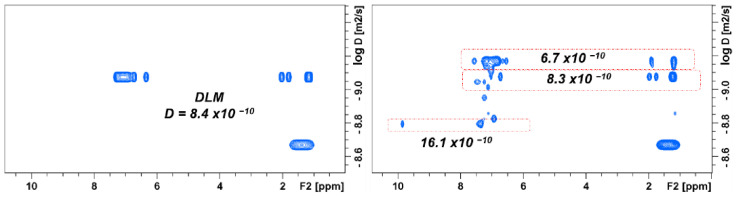
DOSY (600 MHz, C_6_D_12_, 300 K) maps of (left) *a*-DLM compared to (right) stressed sample.

DOSY (Diffusion Ordered SpectroscopY) map of the mixture was compared with that of pure *a*-DLM (Figure 6), for which a diffusion coefficient (D) of 8.4 × 10^−10^ m^2^/s was measured. In the stressed sample, two clusters of signals are found in addition to *a*-DLM and its inactive isomer. One of them is characterized by an average diffusion coefficient of 6.7 × 10^−10^ m^2^/s, indicating the presence of species with larger hydrodynamic radius, whilst for the other, a value of 16.1 × 10^−10^ m^2^/s is measured. The latter diffusion coefficient was attributed to by-product 3-PBA (the diffusion coefficient of the pure aldehyde is reported in Figure S5). The data collected from DOSY experiments does not support the presence of other DLM diastereomers, which are expected to have the same diffusion coefficient measured for *a*-DLM and *i*-DLM.

The HPLC analysis of the mixture (Figure S6) revealed the presence of two further compounds in addition to *a*-DLM, *i*-DLM, and 3-PBA. The five compounds were isolated and, after solvent evaporation, analyzed via NMR in deuterated cyclohexane (Figure 5) and in solution A (Figure S7). The analysis of the single proton spectra allowed identifying *i*-DLM, *a*-DLM, and 3-PBA as the peaks eluting at 9.0, 10.3, and 12.2 min, respectively. The proton spectra of compounds eluting at 11.3 min (Figure 5d) and 12.9 min (Figure 5f), on the contrary, suggest that the two species have a very similar chemical structure. None of the isolated components corresponds to DMA, as expected.

**Scheme 2:**
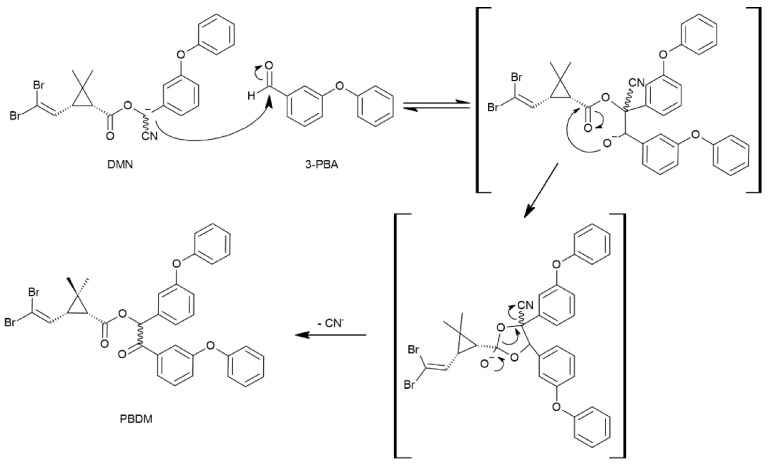
Suggested reaction mechanism involving DLM and PBA, with formation of two PBDM diastereomeric species.

The chemical structure of these compounds was thoroughly investigated via NMR analysis in deuterated cyclohexane, where both species are stable over time (Figure S8), unlike the polar mixture, where they are labile and degrade more quickly (Figure S9). From the comparison of spectra reported in Figure 5d,f (and Figure S7d,f), it can be observed that the high frequency region of the two compounds is very similar, the biggest difference being represented by a doublet resonating at 6.80 and 6.65 ppm, respectively. This signal was attributed to an olefinic proton by analysis of the heteronuclear HSQC (Heteronuclear Single Quantum Correlation) maps (^13^C resonating at 133.8 and 133.2 ppm respectively, Figure S10). The singlets centered at 6.53 and 6.55 ppm correlate with carbons resonating at 78.4/78.3 ppm; if compared to DLM α-benzyl position (^1^H at 6.34/6.28 ppm, ^13^C at 62.8 ppm), these benzylic protons are more deshielded, suggesting their proximity to an additional electron withdrawing group. The examination of the low frequency region between 2.20 and 1.00 ppm indicates that each compound is constituted by one decamethrinic moiety, and quantitative analysis revealed the presence of 18 aromatic protons (Figure S11). Finally, ESI-MS analysis found for both compounds a nominal molecular weight of 674 Da (Figure S12). All the data suggest the occurring of a nucleophilic addition of deltamethrin on 3-PBA with formal loss of cyanide (Scheme 2). This reaction has been reported between DLM and acetone [38], and in the current case, it involves the only carbonyl compound present in the mixture, the aldehyde. Like DLM, the product of this reaction has three chiral centers, two that remain unchanged at the aliphatic ring and one at the benzylic position, whose configuration depends on the direction of the nucleophilic addition, thus generating a diastereomeric pair. This reaction and the related mechanism explain the presence of two species with very similar NMR profile, same diffusion coefficient and same mass. Since these products are the result of a reaction between 3-phenoxybenzaldehyde (3-PBA) and deltamenthrin (DLM), they were named α-3-phenoxybenzoyl-deltamethrin (*R*-PBDM and *S*-PBDM), with the chiral configuration of the benzyl carbon differentiating between the two diastereomers (Scheme 2).

**Figure 7:**
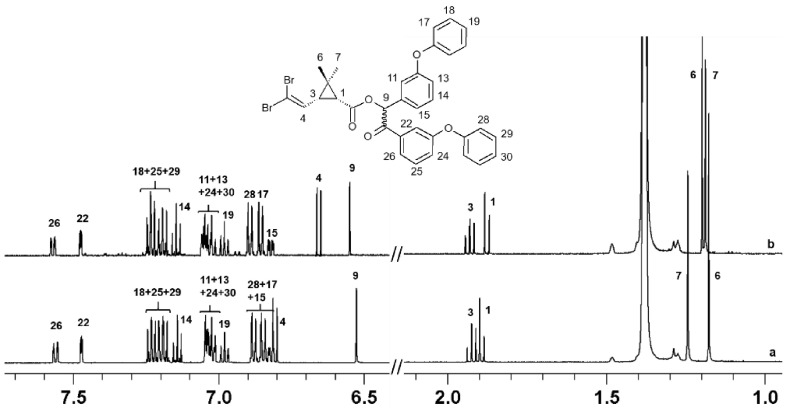
^1^H NMR (600 MHz, C_6_D_12_, 300 K) spectra of (**a**) *R*/*S*-PBDM and (**b**) *S*/*R*-PBDM with numbering scheme for NMR analysis.

The dipolar interactions were analyzed via ROESY (Rotating-frame Overhauser Enhancement SpectroscopY) experiments (see Supplementary Materials), with the aim to match each diastereomer with its NMR profile. Unfortunately, no significant differences were detected, and it was not possible to discriminate between the two compounds. The chiral center 9 is the one involved in the epimerization, nonetheless the chemical shift of the alkenyl proton H4 is the most affected by the change in configuration (|Δδ|H4 = 0.15 ppm, compared to |Δδ|H9 = 0.02 ppm, Figure 7). This effect might be the result of a different spatial arrangement of the aromatic rings in the two diastereomers with respect to the dibromovinyl moiety, which moves H4 to a zone with distinct shielding characteristics.

Overall, the two species show some differences in their ^1^H and ^13^C chemical shifts, but the extent of the differentiation does not allow to discriminate them with the support of molecular modeling, since Δδ falls within the error of the calculations. Further studies therefore are needed in order to optimize the synthetic procedure and the purification step, so that the compounds are recovered in sufficient amounts to guarantee a full analysis. 

For what concerns the scope of this study, it would be interesting to evaluate the toxicological and insecticidal impact of PBDM presence, since it could affect the performance of IPs (or of analogous formulations) containing deltamethrin as active insecticide. As the data show, over time, *a*-DLM concentration significantly decreases in alkaline environment. DMA and 3-PBA are the major by-product resulting from chemical degradation, with the latter contributing in further reducing DLM concentration (of both active and inactive diastereomer) by leading to PBDM formation.

## 3. Materials and Methods

### 3.1. Materials and Reagents

Active deltamethrin (*a*-DLM), 3-phenoxybenzaldehyde (3-PBA), deuterated acetonitrile (ACN-d_3_), deuterated cyclohexane (C_6_D_12_), sodium tetraborate (Na_2_B_4_O_7_), and hydrochloric acid (HCl 36%) were purchased from Sigma Aldrich (St. Louis, MI, USA) and employed without further purification. *cis*-Decamethrinic acid (DMA) was purchased from BOC Sciences (Shirley, New York, NY, USA). Hexane and isopropanol (IPA) were purchased from Thermo Fisher Scientific (Waltham, MA, USA). The reference mixture of eight deltamethrin stereoisomers was purchased from Bayer (Frankfurt am Main, Germany).

### 3.2. Samples Preparation

#### 3.2.1. Solution A

The 18.58 mg of *a*-DLM (3.68 × 10^−2^ mmol) were dissolved into 2.73 g of ACN-d_3_, followed by addition of demineralized water (0.74 g, pH 8) to reach an approximate 80:20 ACN/H_2_O weight ratio and a final concentration of 9.25 mM for DLM (0.5% wt). The buffered water was prepared by mixing 1.17 mL of Na_2_B_4_O_7_ 0.05 M to 0.88 mL of HCl 0.1 M.

#### 3.2.2. Solution B

The 21.07 mg of *a*-DLM (4.17 × 10^−2^ mmol) were dissolved into 3.10 g of ACN-d_3_, followed by addition demineralized water (0.87 g, pH 9) to reach an approximate 80:20 ACN/H_2_O weight ratio and a final concentration of 9.18 mM for DLM (0.5% wt). The buffered water was prepared by adding 1.71 mL of Na_2_B_4_O_7_ 0.05 M to 0.29 mL HCl 0.1 M.

### 3.3. HPLC Analysis

High performance liquid chromatography (HPLC) experiments were carried out on an Agilent 1200 high-performance liquid chromatograph (Agilent Technology, Santa Clara, CA, USA) equipped with a G1322A degasser, a G1310A isocratic pump, a G1314B UV detector set at 225 nm, a G1328B manual injector of 20 μL and an Agilent 1200 chemical analytical workstation. Chromatographic separation was conducted at 25 °C, and the flow rate was maintained at 1.0 mL/min. Chiral analysis was conducted according to the enantioselective HPLC method CIPAC/4907 provided from Bayer, using a Chiralcel OD-H column (Daicel Chemical Industries Ltd. Japan, 250 × 4.6 mm i.d., 5 μm particle size), protected with a guard column of the same phase. A mixture of *n*-hexane/isopropanol (95:5 *v*/*v*) was used as eluent, whereas the samples were dissolved in *n*-hexane [39].

### 3.4. MS Analysis

Flow Injection ESI-MS measurements were performed on a Waters Xevo Q-ToF (Milford, MA, USA), operated in the ESI-positive ion mode. The samples were dissolved in methanol and 50 µL were injected into a 50 µL/min methanol flow that was led to the ESI source. The MS conditions were as follows: capillary voltage 3000 V, sample cone voltage 45 V, desolvation temperature 150 °C, source temperature 80 °C, desolvation gas flow 400 L/h.

### 3.5. NMR Analysis

Nuclear Magnetic Resonance (NMR) measurements were performed on a Bruker AVANCE III HD and a Bruker AVANCE NEO spectrometer (Billerica, MA, USA), equipped with a 5 mm BBO Prodigy™ cryoprobe and a 5 mm BBI probe respectively, both operating at 600 MHz for ^1^H and 150 MHz for ^13^C nuclei, respectively. All data were processed using the Topspin software versions 3.6.2 or 4.0.9. The experimental details of NMR measurements are reported in the Supplementary Materials.

### 3.6. Characterization of R-PBDM and S-PBDM

#### 3.6.1. R/S-PBDM

^1^H NMR (600 MHz, C_6_D_12_, 300 K): δ (ppm) = 7.56 (ddd, ^3^*J*_26-25_ = 7.8 Hz, ^4^*J*_26-22_ = 1.6 Hz, ^4^*J*_26-24_ = 1.0 Hz, 1H, H26), 7.47 (dd, ^4^*J*_22-24_ = 2.4 Hz, ^4^*J*_22-26_ = 1.6 Hz, 1H, H22), 7.25-7.16 (m, 5H, H25 + H29 + H18), 7.14 (t, ^3^*J*_14-15_ = ^3^*J*_14-13_ = 8.1 Hz, 1H, H14), 7.05-7.03 (m, 2H, H11 + H24), 7.02 (ddd, ^3^*J*_13-14_ = 8.1 Hz, ^4^*J*_13-15_ = 2.4 Hz, ^4^*J*_13-11_ = 1.0 Hz, 1H, H13), 7.02 (t, ^3^*J*_30-29_ = 7.4 Hz, 1H, H30), 6.98 (t, ^3^*J*_19-18_ = 7.5 Hz, 1H, H19), 6.88 (m, 2H, H28), 6.85 (m, 2H, H17), 6.82 (ddd, ^3^*J*_15-14_ = 8.1 Hz, ^4^*J*_15-13_ = 2.4 Hz, ^4^J_15-11_ = 1.0 Hz, 1H, H15), 6.80 (d, ^3^*J*_4-3_ = 8.2 Hz, 1H, H4), 6.53 (s, 1H, H9), 1.92 (t, ^3^*J*_3-4_ = ^3^*J*_3-1_ = 8.2 Hz, 1H, H3), 1.89 (d, ^3^*J*_1-3_ = 8.2 Hz, 1H, H1), 1.24 (s, 3H, H6), 1.18 (s, 3H, H7). ^13^C NMR (150 MHz, C_6_D_12_, 300 K): δ (ppm) = 192.1 (C20), 169.8 (C8), 158.6 (C23, C12), 157.6 (C16), 157.4 (C27), 137.2 (C21), 136.8 (C10), 133.8 (C4), 130.4 (C14), 130.3 (C29), 130.1-130.0 (C18, C25), 124.3 (C30), 123.8 (C19), 123.7 (C11,C24), 123.6 (C26), 123.2 (C13), 120.0 (C11, C24), 119.5 (C15, C17, C28), 119.0 (C22), 90.1 (C5), 78.4 (C9), 36.7 (C3), 32.1 (C1), 28.7 (C6), 28.5 (C2), 15.1 (C7). HPLC (Chiralcel OD-H, 1.0 mL/min, *n*-hexane/isopropanol 95:5 *v*/*v*, 225 nm): T_R_ = 11.3 min. MS (TOF MS ES^+^) *m*/*z* found for C_34_H_28_Br_2_O_5_Na [M + Na]^+^ 699.0. Nuclei are numbered according to Figure 7.

#### 3.6.2. S/R-PBDM

^1^H NMR (600 MHz, C_6_D_12_, 300 K): δ (ppm) = 7.57 (ddd, ^3^*J*_26-25_ = 7.8 Hz, ^4^*J*_26-22_ = 1.5 Hz, ^4^*J*_26-24_ = 1.1 Hz 1H, H26), 7.47 (dd, ^4^*J*_22-24_ = 2.4 Hz, ^4^*J*_22-26_ = 1.5 Hz, 1H, H22), 7.26-7.17 (m, 5H, H25 + H29 + H18), 7.15 (t, ^3^*J*_14-15_ = 8.2 Hz, 1H, H14), 7.07-7.00 (m, 4H, H11 + H13 + H24 + H30), 6.98 (t, ^3^*J*_19-18_ = 7.4 Hz, 1H, H19), 6.89 (m, 2H, H28), 6.86 (m, 2H, H17), 6.82 (ddd, ^3^*J*_15-14_ = 8.2 Hz, ^4^*J*_15-11_/^4^*J*_15-13_ = 2.3 Hz/1.0 Hz, 1H, H15), 6.65 (d, ^3^*J*_4-3_ = 8.3 Hz, 1H, H4), 6.55 (s, 1H, H9), 1.93 (t, ^3^*J*_3-4_ = ^3^*J*_3-1_ = 8.3 Hz, 1H, H3), 1.87 (d, ^3^*J*_1-3_ = 8.3 Hz, 1H, H1), 1.20 (s, 3H, H6/H7), 1.19 (s, 3H, H7/H6). ^13^C NMR (150 MHz, C_6_D_12_, 300 K): δ (ppm) = 191.3 (C20), 169.5 (C8), 159.1 (C23, C12), 158.2 (C16) 157.2 (C27), 137.4 (C21), 136.8 (C10), 133.2 (C4), 130.5 (C14), 130.2 (C29), 130.0 (C18, C25), 124.3 (C30, C19), 123.6 (C26), 123.5 (C11, C24), 123.2 (C13), 120.0 (C11, C24), 119.3 (C15, C17, C28), 118.8 (C22), 90.4 (C5), 78.3 (C9), 36.1 (C3), 32.1 (C1), 28.7 (C6/C7), 28.5 (C2), 15.1 (C7/C6). HPLC (Chiralcel OD-H, 1.0 mL/min, *n*-hexane/isopropanol 95:5 *v*/*v*, 225 nm): T_R_ = 12.0 min. MS (TOF MS ES^+^) *m*/*z* found for C_34_H_28_Br_2_O_5_Na [M + Na]^+^ 699.0. Nuclei are numbered according to Figure 7.

## 4. Conclusions

The aim of the present study was the elucidation of active deltamethrin degradation profile in an environment whose solvent composition mimics a water-based insecticidal paint. The thorough NMR analysis, performed on the insecticide over a four-week period, allowed the identification of a new pair of diastereomer by-products, which were characterized by combining NMR, HPLC, and MS analyses. Further studies focused on the elucidation of the chiral configuration are planned in the future.

The investigation carried out gives new insights into the insecticide degradation routes and might open the way to studies focused on determining the insecticidal and toxicological influence exerted by the by-products, leading ultimately to an optimization of the insecticidal formulation, and hence into a better product. It is important to underline that the reaction behind PBDM formation implicates the consumption of the 3-phenoxybenzaldehyde, known for its toxicity, even if at the expense of the insecticide.

## Figures and Tables

**Table 1:** DLM degradation products reported in the literature.

Name	Chemical Structure
2-OH deltamethrin	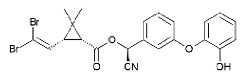
4-OH deltamethrin	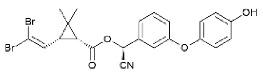
α-hydroxy-3-phenoxy-benzeneacetonitrile	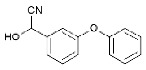
3-phenoxybenzaldehyde	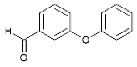
2-hydroxy-4-methoxy benzophenone	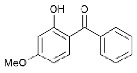
3-phenoxybenzoic acid	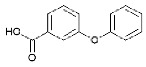
*cis*-decamethrinic acid	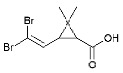

## Data Availability

The data presented in this study are available in the Supplementary materials.

## Supplementary Materials

The following are available online, NMR experimental, Figure S1: ^1^H NMR spectrum of *a*-DLM, Figure S2: HPLC chromatogram, Table S1: Proton chemical shift measured for *a*-DLM and *i*-DLM, Figure S3: ^1^H NMR spectra of *a*-DLM in ACN-d_3_/H_2_O, solution A and solution B, Table S2: Quantitative composition of *a*-DLM (solution A) at room temperature, Table S3: Quantitative composition of *a*-DLM (solution B) at room temperature, Figure S4: Degradation of *a*-DLM at room temperature in solution A, Table S4: Quantitative composition of *a*-DLM (solution A) at 40 °C, Table S3: Quantitative composition of *a*-DLM (solution B) at 40 °C, Figure S5: DOSY map of 3-PBA, Figure S6: HPLC chromatogram of *a*-DLM stressed sample, Figure S7: ^1^H NMR spectra of stressed sample and compounds isolated from fraction collection, Figure S8: ^1^H NMR spectra in cyclohexane over time, Figure S9: ^1^H NMR spectra in ACN-d_3_/H_2_O over time, Figure S10: HSQC maps, Figure S11: ^1^H NMR quantitative spectrum, Figure S12: TOF MS-ESI^+^ spectra, Figure S13: GC-MS chromatograms, Figure S14: EI-MS spectra, Figure S15: ^1^H NMR spectra comparison, Characterization data and 2D NMR maps.
