# Supplementary material for: New Insights into the Degradation Path of Deltamethrin
Source: Molecules. 2021 Jun 22;26(13):3811. doi: 10.3390/molecules26133811 (PMC8270271; doi:10.3390/molecules26133811)
Supplement: Supplementary file 1 [file molecules-26-03811-s001.zip › molecules-1256776-supplementary.pdf]

New Insights into the Degradation Path of Deltamethrin

Federica Aiello [1,2,*], Marcel G. Simons [1], Jan W. van Velde [1] and Paulo Dani [1,*]

Supplementary Materials

Table of contents

- NMR experimental 2
- Fig S1 3
- Preparation of inactive deltamethrin and chromatographic separation 3
- Fig S2 3
- Table S1 4
- Fig S3 4
- Table S2 4
- Table S3 5
- Fig S4 5
- Table S4 5
- Table S5 5
- Fig S5 6
- Fig S6 6
- Fig S7 6
- Fig S8 7
- Fig S9 7
- Fig S10 7
- Fig S11 8
- Identification of DMA and 3-PBA 8

  Fig S12 8

  Fig S13 9

  Fig S14 9

  Fig S15 10
- Characterization data 10

NMR experimental

Analysis of *a*-DLM degradation was performed in ACN-d_3/H_2O (solution A and solution B) by running quantitative proton spectra with 30 s of relaxation delay (d1), and a 90 degrees pulse of 9.3 μs. 16 Scans and a spectral width of 20 ppm were used, the FID was acquired into 64K data points during a 2.7 s acquisition time. Resulting data were Fourier transformed after multiplying by exponential window function using a line broadening function of 0.3 Hz. The isolated by-products were characterised in C_6D_{12}, by optimising the parameters settings (d1, number of scans, 90 degrees pulse) for each sample. Resulting data were Fourier transformed after multiplying by exponential window function using a line broadening function of 0.3 Hz.

DOSY (Diffusion Ordered SpectroscopY) experiments were performed on samples dissolved in C_6D_{12} by using the Bruker pulse sequence dstegp2s_2d. 32 scans and a d1 of 2 s were used, 90 degrees pulse and spectral width were optimized for each sample, the FID was acquired into 32K data points during a 2.1 s acquisition time. The diffusion time (Δ, d20) was set at 150 ms with a gradient length (δ, p30) of 1 ms. Diffusion data were processed with Dynamics Centre 2.2.1 (Bruker).

2D experiments performed on DMN by-products were run in C_6D_{12} using the following parameters:

HSQC (Heteronuclear Single Quantum Correlation) - number of scans optimised for each sample, d1 of 1.5 s, FID acquired into 2K x256 data points during a 0.1 s acquisition time. CNST2 ($^1J_{(CH)}$ coupling constant) was set on 145.0 Hz (Bruker pulse program: hsqcedetgpsisp2.3);

HMBC (Heteronuclear Multiple Bond Coherence) - number of scans optimised for each sample, d1 of 1.5 s, FID acquired into 8Kx128 data points during a 0.1 s acquisition time. CNST2 ($^1J_{(CH)}$ coupling constant) and CNST13 ($^nJ_{(CH)}$ long range coupling constant) were set on 145.0 Hz and 8 Hz, respectively (Bruker pulse program: hmbcgplpndqf);

ROESY (Rotating frame Overhauser Enhancement SpectroscopY) - 16 scans, d1 of 2 s, FID acquired into 2Kx256 data points during a 0.2 s acquisition time. Spinlock (P15) set on 0.3 and 0.6 s (Bruker pulse program: roesyphpp.2).

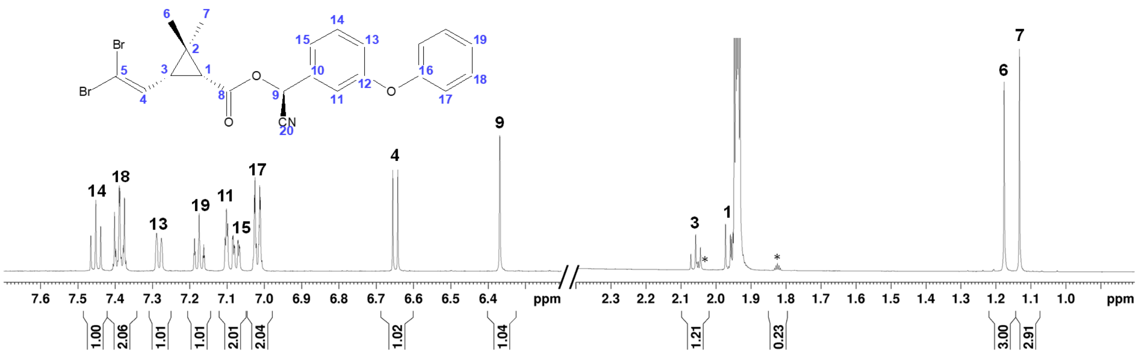

Figure S1. ^{1}H NMR (600 MHz, ACN-d_3/H_2O 80:20 m/m, 300 K) spectrum of *a*-DLM (9.2 mM) with peaks attribution. * indicate ACN satellites.

<u>Preparation of inactive deltamethrin and chromatographic separation</u>

The low stability of active deltamethrin towards alcohols was exploited for generating its inactive diastereomer. *a*-DLM was dissolved in isopropanol (final concentration 8.3 mM) and left under stirring for one week, then the solution was analysed via HPLC. The chromatogram reported in **Figure S2** shows the peak eluting at around 10.1 min belonging to *a*-DLM, plus another one (9.5 min) with similar intensity. This peak was attributed to *i*-DLM by comparison with the reference mixture of the eight different enantiomers. Two minor peaks eluted around 11.4 min and 16 min, but their concentration was too low for the isolation and characterisation of the corresponding species. *i*-DLM was easily isolated via chromatographic fraction collection and its NMR spectrum recorded in the same solvent mixture used for *a*-DLM (ACN-d_3/H_2O, 80:20 m/m) to compare the NMR profile of the two diastereomers (see main text).

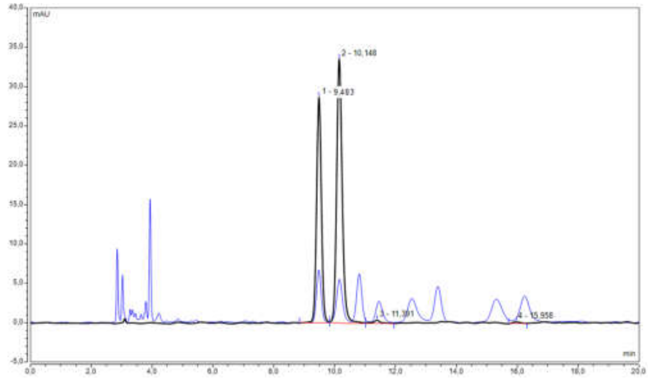

Figure S2. HPLC chromatogram of (black) stressed *a*-DLM compared with (blue) the reference mixture of DLM enantiomers.

Table S1. Proton chemical shift (δ, ppm) measured for *a*-DLM and *i*-DLM in ACN-d3/H_2O 80:20 m/m and chemical shift differences (Δδ, Hz).

proton	δ (ppm)		Δδ (*i*-DLM − *a*-DLM) (Hz)
	a-DLM	*i*-DLM	
1	1.97	1.96	-7.4
3	2.06	2.04	-12.7
4	6.65	6.62	-14.3
6	1.18	1.21	20.0
7	1.13		46.3
9	6.37	6.35	-9.7
11	7.10	7.11	7.6
13	7.28	7.29	1.8
14	7.45	7.45	1.4
15	7.08	7.08	-0.1
17	7.02	7.02	1.1
18	7.39	7.39	3.4
19	7.17	7.18	2.1

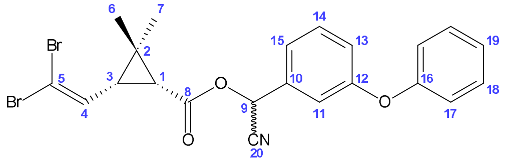

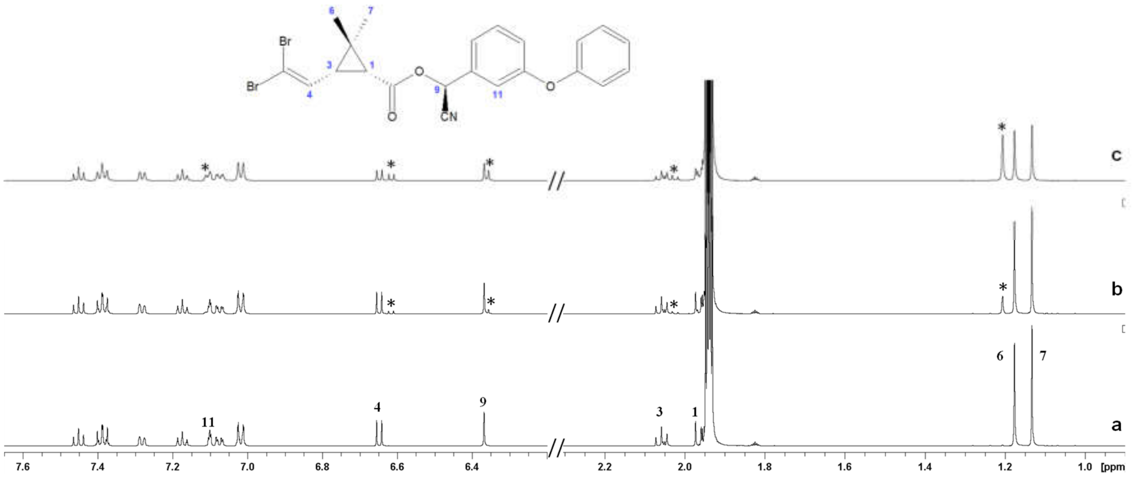

Figure S3. ¹H NMR (600 MHz, 300 K) spectra of *a*-DLM (9.2 mM) recorded right after sample preparation (at room temperature) in (a) ACN-d3/H_2O 80:20 m/m, (b) solution A and (c) solution B. The symbol (*) indicates resonances belonging to *i*-DLM; the molecular structure on the top belongs to *a*-DLM and the most differentiated protons between the diastereomers are numbered.

Table S2. Quantitative composition (NMR data) of *a*-DLM (solution A) over time at room temperature.

t (days, d)	*a*-DLM (%)	*i*-DLM (%)	3-PBA[a] (%)	DMA[b] (%)	*R*/*S*-PBDM[c] (%)	*S*/*R*-PBDM[c] (%)
t0	87.5	12.5				
1d	48.8	51.2				
2d	45.7	50.1	2.0	2.2		
3d	43.9	50.3	2.0	2.8	0.5	0.5
4d	43.6	49.8	2.2	3.3	0.5	0.5
5d	42.8	49.9	2.2	3.7	0.7	0.7
6d	42.6	48.7	2.2	4.7	0.9	0.9
7d	42.2	48.6	2.7	4.5	1.0	1.0
11d	40.0	46.2	3.2	7.7	1.5	1.5
14d	39.3	44.5	3.7	8.7	1.9	1.9
28d	36.0	41.2	4.8	12.0	3.0	3.0

a: 3-phenoxybenzaldehyde; b: *cis*-decamethrinic acid; c: α-phenoxybenzyl deltamethrin.

Table S3. Quantitative composition (NMR data) of *a*-DLM (solution B) over time at room temperature.

t (days, d)	*a*-DLM (%)	*i*-DLM (%)	3-PBA[a] (%)	DMA[b] (%)	*R*/*S*-PBDM[c] (%)	*S*/*R*-PBDM[c] (%)
t0	62.3	37.7				
1d	49.1	50.9				
2d	43.4	48.2	2.1	4.4	0.9	0.9
3d	41.3	46.8	2.3	6.5	1.6	1.6
4d	40.0	46.4	3.1	7.5	1.5	1.5
5d	38.5	45.6	3.8	8.4	1.8	1.8
6d	37.8	44.5	3.8	9.2	2.3	2.3
7d	37.9	44.5	3.8	9.2	2.3	2.3
11d	33.4	41.7	4.3	10.3	3.4	2.3
14d	30.3	36.4	5.1	17.9	5.1	5.1
28d	23.4	27.6	6.9	27.2	7.5	7.5

a: 3-phenoxybenzaldehyde; b: *cis*-decamethrinic acid; c: α-phenoxybenzyl deltamethrin.

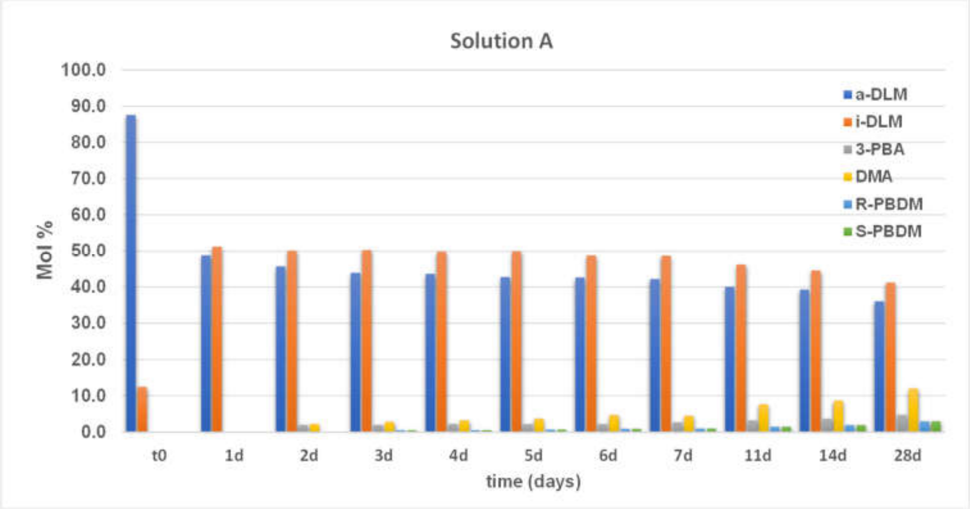

Figure S4. Degradation of *a*-DLM over time at room temperature in solution A. DLM: deltamethrin; 3-PBA: 3-phenoxybenzaldehyde; DMA: *cis*-decamethrinic acid; PBDM: α-3-phenoxybenzoyl-deltamethrin.

Table S4. Quantitative composition (NMR data) of *a*-DLM in solution A kept at 40°C for the indicated time period.

t (days, d)	*a*-DLM[a] (%)	*i*-DLM[a] (%)	3-PBA[a] (%)	DMA[a] (%)	*R*-PBDM[a] (%)	*S*-PBDM[a] (%)
5d	30.9	34.8	7.6	18.1	4.3	4.3
11d	28.5	33.1	7.3	20.1	5.5	5.5
28d	27.3	29.8	8.1	22.7	6.1	6.1

[a] DLM: deltamethrin; 3-PBA: 3-phenoxybenzaldehyde; DMA: *cis*-decamethrinic acid; PBDM: α-3-phenoxybenzoyl-deltamethrin.

Table S5. Quantitative composition (NMR data) of *a*-DLM in solution B kept at 40°C for the indicated time period.

t (days, d)	*a*-DLM[a] (%)	*i*-DLM[a] (%)	3-PBA[a] (%)	DMA[a] (%)	*R*-PBDM[a] (%)	*S*-PBDM[a] (%)
5d	8.9	10.9	10.3	45.4	12.2	12.2
11d	3.3	3.4	11.5	58.0	11.9	11.9
28d	1.2	1.2	14.2	60.6	11.4	11.4

[a] DLM: deltamethrin; 3-PBA: 3-phenoxybenzaldehyde; DMA: *cis*-decamethrinic acid; PBDM: α-3-phenoxybenzoyl-deltamethrin.

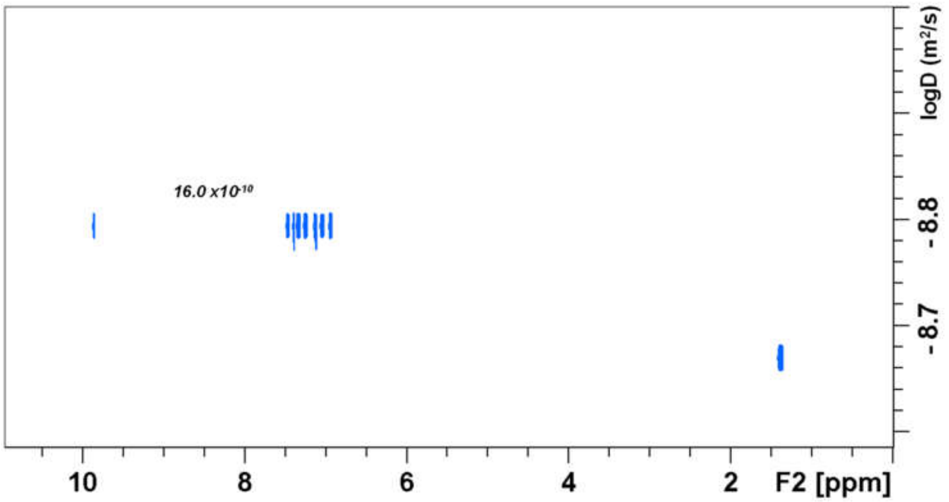

Figure S5. DOSY (600 MHz, C_6D_{12}, 300 K) map of 3-PBA.

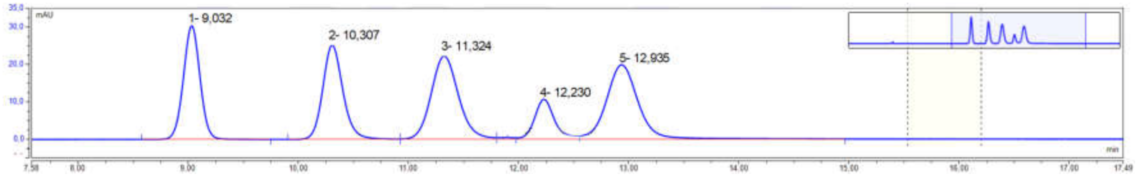

Figure S6. HPLC chromatogram of *a*-DLM stressed sample (eluent mixture: n-hexane:IPA 95:5 v/v).

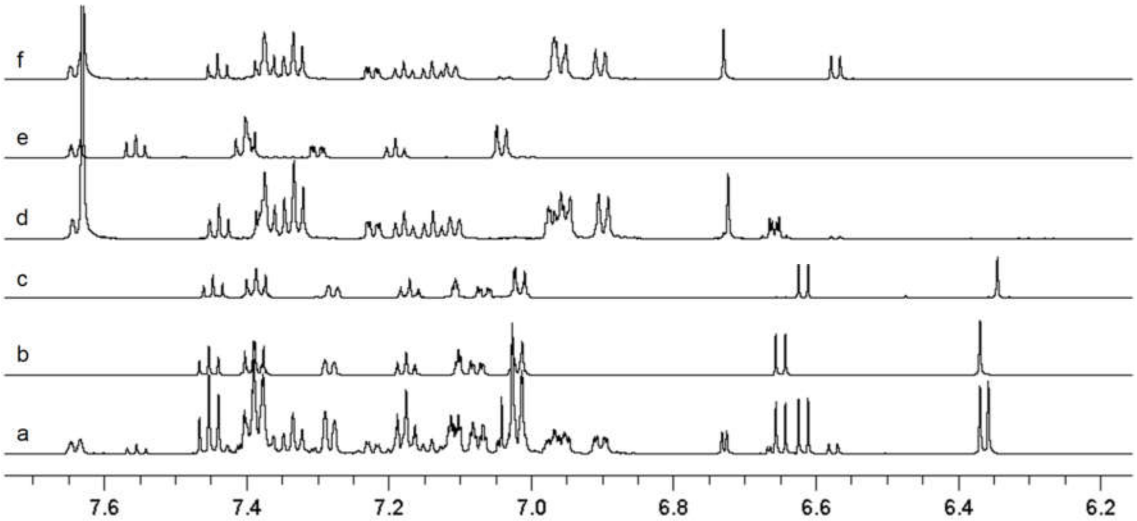

Figure S7. ^{1}H NMR (600 MHz, solution A, 300 K) spectra of (a) stressed sample and compounds isolated from fraction collection: (b)*i*-DLM (9.0 min), (c) *a*-DLM (10.3 min), (d) *S*-PBDM (11.3 min), (e) 3-PBA (12.2 min), and (f) *R*-PBDM (12.9 min).

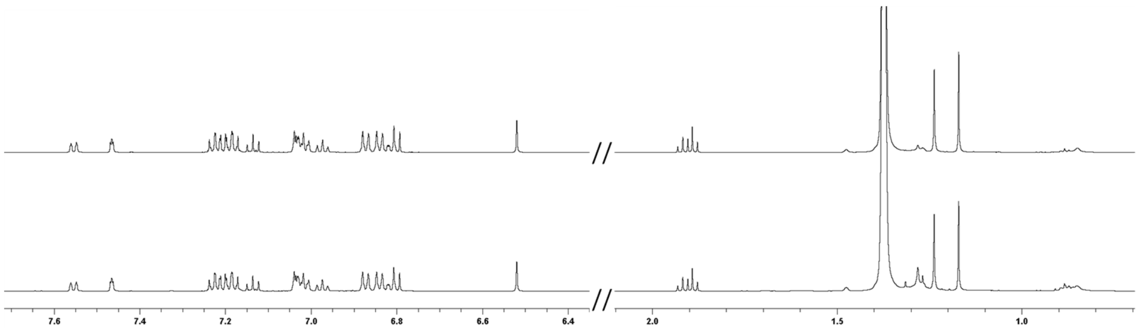

Figure S8. ^{1}H NMR (600 MHz, C_6D_{12}, 300 K) spectra of the compound eluting at 11.3 min (top) freshly dissolved in the solvent and (bottom) after 2 years from sample preparation.

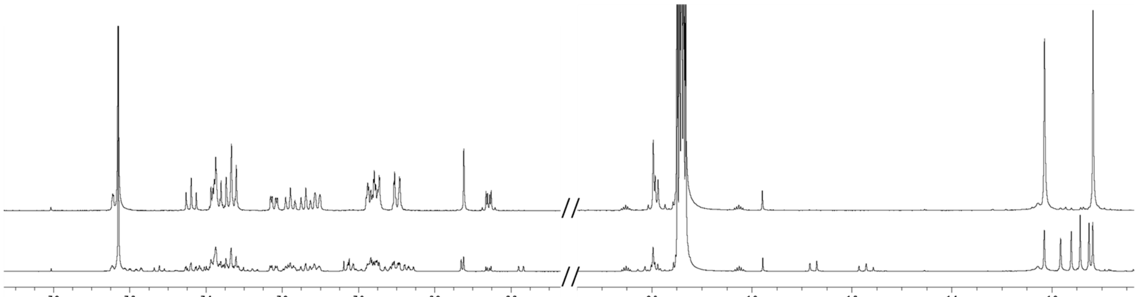

Figure S9. ^{1}H NMR (600 MHz, solution A, 300 K) spectra of the compound eluting at 11.3 min (top) freshly dissolved in the solvent mixture and (bottom) after 2 months from sample preparation.

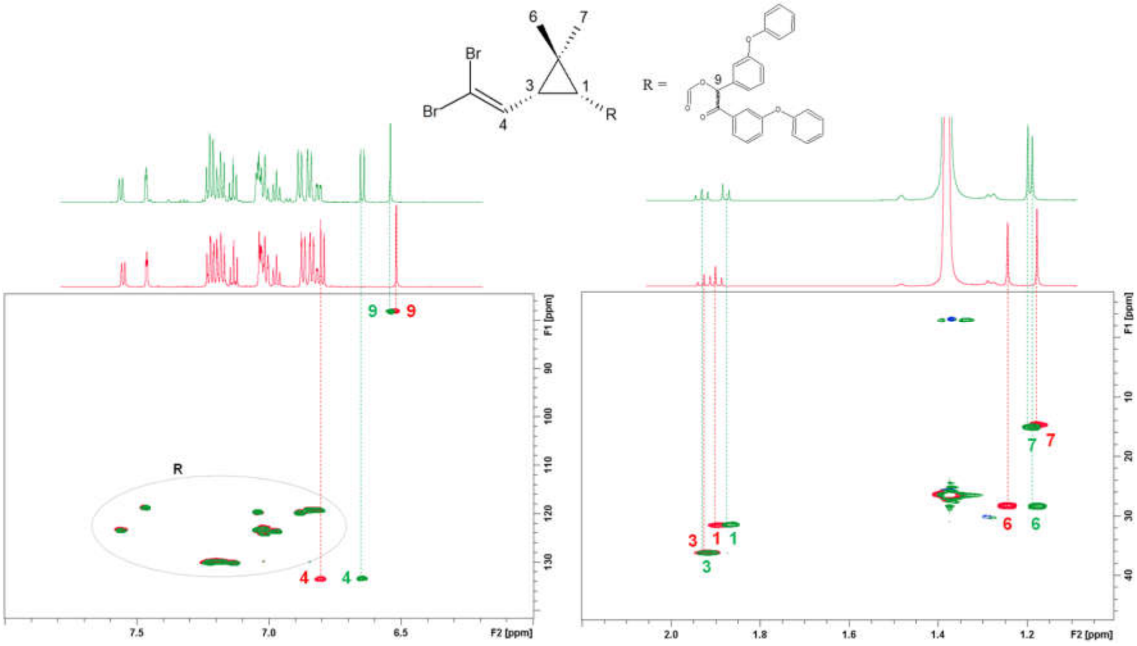

Figure S10. Expansions of HSQC (600 MHz, C_6D_{12}, 300 K) maps of (red) compound eluting at 11.3 min and (green) compound eluting at 12.9 min.

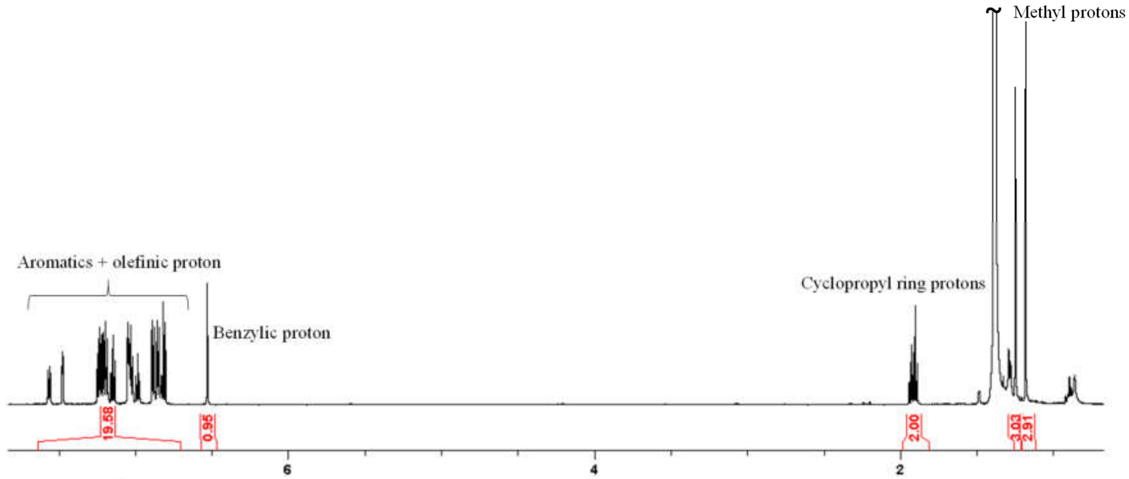

Figure S11. ^{1}H NMR (600 MHz, C_6D_{12}, 300 K) spectrum of the compound eluting at 11.3 min with integration.

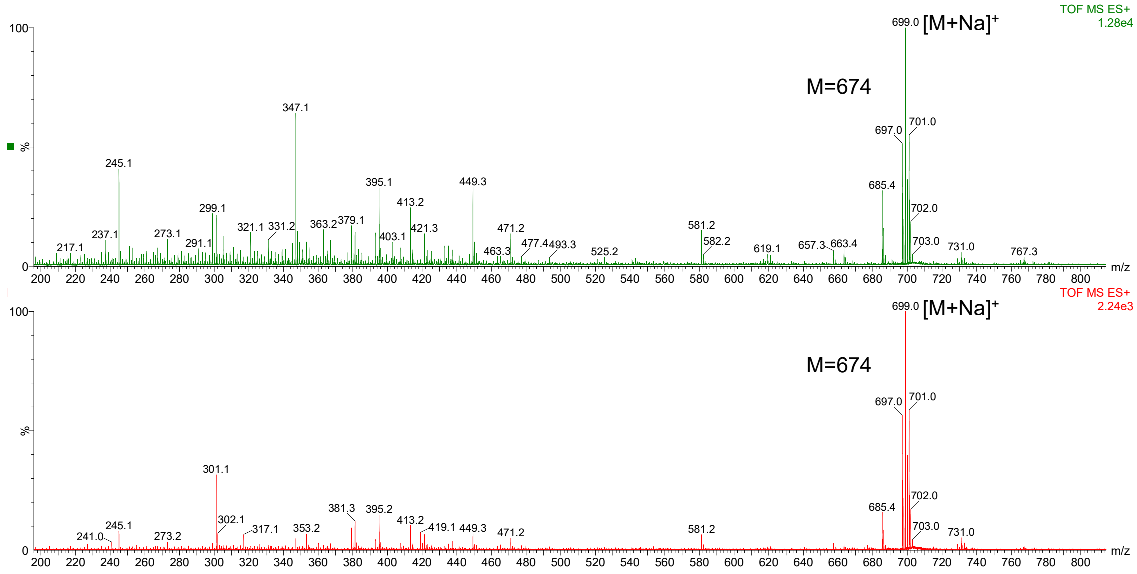

Figure S12. TOF MS ESI$^+$ spectra of (red, bottom) compound eluting at 11.3 min and (green, top) compound eluting at 12.9 min.

Identification of DMA and 3-PBA

The identification of the two by-products was performed on a portion of solution B kept at 40 ºC for four weeks. 100 μL of sample were dried, dissolved in hexane and submitted to GC-MS analysis. Figure S13 compares the chromatogram of the stressed sample with the one of a freshly prepared *a*-DLM solution. Here, the most intense peak eluting at 16.5 min belongs to *a*-DLM; another peak eluting at 16.4 min is found, which corresponds to the diastereomer *i*-DLM, most likely formed by thermally induced degradation in the GC injection system. The same peaks are found in the chromatogram of the stressed solution, together with other two peaks eluting at 8.5 min and 9.0 min, identified by MS as *cis*-decamethrinic acid (DMA) and the aldehyde 3-PBA (Figure S14), respectively.

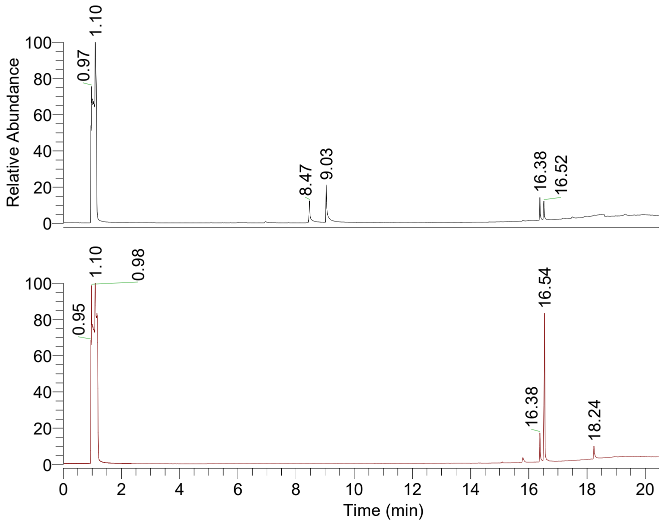

Figure S13. GC-MS total ion content (TIC) chromatograms of *a*-DLM sample (bottom) freshly prepared and (top) after 28 days at 40ºC in solution B.

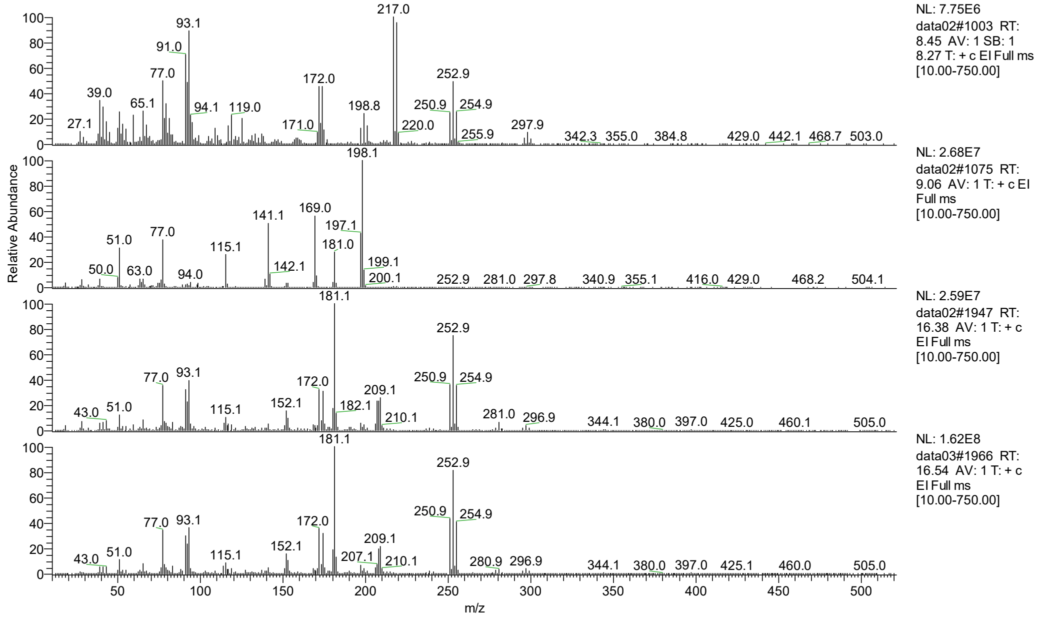

Figure S14. EI-MS spectra, from top to bottom: DMA, 3-PBA, *i*-DLM and *a*-DLM.

As further confirmation, the proton spectrum of the 28 day stressed solution was compared with those of pure DMA and 3-PBA (Figure S15).

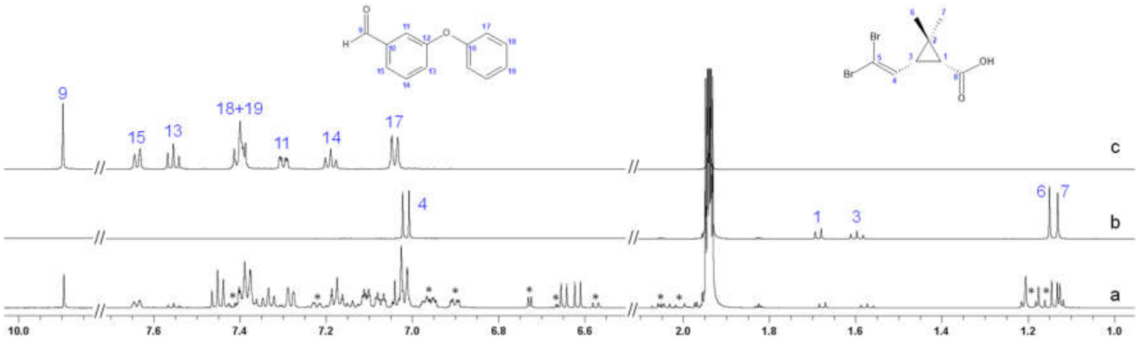

Figure S15. ^{1}H NMR (600 MHz, ACN-d$_3$/H$_2$O 80:20 m/m, 300 K) spectra of (a) *a*-DLM after 28 days from preparation at 40ºC, (b) DMA, (c) 3-PBA with peaks attribution. * indicate resonances belonging to additional by-products.

<u>Characterisation data</u>

3-PBA (numbering scheme reported in **Figure S15**)

^{1}H NMR (600 MHz, C_6D_{12}, 300 K): δ (ppm) = 9.86 (s, 1H, H9), 7.46 (dt, $^{3}J_{15\text{-}14}$ = 7.6 Hz, $^{4}J_{15\text{-}10}$ = $^{4}J_{15\text{-}13}$ = 1.2 Hz, 1H, H15), 7.39 (dd, $^{4}J_{11\text{-}15}$ = 1.2 Hz, $^{4}J_{11\text{-}13}$ = 2.4 Hz, 1H, H11), 7.33 (t, $^{3}J_{14\text{-}13}$ = $^{3}J_{14\text{-}15}$ =7.6 Hz, 1H, H14), 7.25 (t, $^{3}J_{18\text{-}17}$ = $^{3}J_{18\text{-}19}$ =7.5 Hz,2H, H18), 7.12 (ddd, $^{3}J_{13\text{-}14}$ = 7.6 Hz, $^{4}J_{13\text{-}11}$ = 2.4 Hz, $^{4}J_{13\text{-}15}$ = 1.2 Hz, 1H, H13), 7.04 (tt, $^{3}J_{19\text{-}18}$ = 7.5 Hz, $^{4}J_{19\text{-}17}$ = 1.1 Hz, 1H, H19), 6.93 (dd, $^{3}J_{17\text{-}18}$ = 7.5 Hz, $^{3}J_{17\text{-}19}$ = 1.1 Hz, 2H, H17). ^{13}C NMR (150 MHz, C_6D_{12}, 300 K): δ (ppm) = 188.8 (C9), 159.4 (C12), 157.5 (C16), 139.7 (C10), 130.4 (C14), 130.3 (C18), 124.4 (C19), 124.3 (C15), 124.2 (C13), 119.9 (C17), 118.9 (C11).

HPLC (Chiralcel OD-H, 1.0 mL/min, n-hexane/isopropanol 95:5 v/v, 225 nm): T_R = 12.2 min.

MS: m/z 198 (M^+, 100%), 169 (59), 141 (49), 197 (42), 77 (36).

a-DMN (numbering scheme reported in **Table S*1***)

^{1}H NMR (600 MHz, C_6D_{12}, 300 K): δ (ppm) = 7.25 (m, 3H, H18 + H14), 7.19 (dt, $^{3}J_{15\text{-}14}$ = 7.7 Hz, $^{4}J_{15\text{-}11}$ = $^{4}J_{15\text{-}13}$ = 2.0 Hz, 1H, H15), 7.10 (t, $^{4}J_{11\text{-}13}$ = $^{4}J_{11\text{-}15}$ = 2.0 Hz, 1H, H11), 7.03 (tt, $^{3}J_{19\text{-}18}$ = 7.4Hz, $^{4}J_{19\text{-}17}$ = 1.1 Hz, 1H, H19), 6.93 (m, 3H, H17 + H13), 6.72 (d, $^{3}J_{4\text{-}3}$ = 8.3 Hz, 1H, H4), 6.34 (s, 1H, H9), 2.02 (t, $^{3}J_{3\text{-}1}$ = $^{3}J_{3\text{-}4}$ = 8.3 Hz, 1H, H3), 1.79 (d, $^{3}J_{1\text{-}3}$ = 8.3 Hz, 1H, H1), 1.18 (s, 3H, H6), 1.14 (s, 3H, H7). ^{13}C NMR (150 MHz, C_6D_{12}, 300 K): δ (ppm) = 168.6 (C8), 159.3 (C12), 157.4 (C16), 135.6 (C10), 132.5 (C4), 130.7 (C14/C18), 130.3 (C14/C18), 124.4 (C19), 122.4 (C15), 120.2 (C17/C13), 120.0 (C17/C13), 118.4 (C11), 115.2 (C20), 91.9 (C5), 62.8 (C9), 37.2 (C3), 31.5 (C1), 28.7 (C2), 28.4 (C6), 15.1 (C7).

HPLC (Chiralcel OD-H, 1.0 mL/min, n-hexane/isopropanol 95:5 v/v, 225 nm): T_R = 10.3 min.

MS: m/z 181 (M^+, 100%), 253 (87), 251 (46), 255 (45), 93 (39).

i-DMN (numbering scheme reported in **Table S*1***)

^{1}H NMR (600 MHz, C_6D_{12}, 300 K): δ (ppm) = 7.25 (3H, H18+H14), 7.15 (1H, H15), 7.12 (1H, H11), 7.04 (1H, H19), 6.94 (2H, H17), 6.93 (H13), 6.69 (1H, H4), 6.28 (1H, H9), 1.98 (1H, H3), 1.79 (1H, H1), 1.28 (3H, H6), 1.23 (3H, H7).

HPLC (Chiralcel OD-H, 1.0 mL/min, n-hexane/isopropanol 95:5 v/v, 225 nm): T_R = 9.0 min.

MS: m/z 181 (M^+, 100%), 253 (79), 77 (42), 93 (42), 251 (40).

DMA (numbering scheme reported in **Figure S15**)

^{1}H NMR (600 MHz, ACN/H_2O 80:20 m/m (water pH 9), 300 K): δ (ppm) = 7.01 (d, $^3J_{4\text{-}3}$ = 8.6 Hz, 1H, H4), 1.68 (d, $^3J_{1\text{-}3}$ = 8.3 Hz, 1H, H1), 1.60 (t, $^3J_{3\text{-}1}$ = $^3J_{3\text{-}4}$ = 8.3 Hz, 1H, H3), 1.15 (s, 3H, H6), 1.13 (s, 3H, H7).

MS:m/z 217 (M^+, 100%), 219 (94), 93 (85), 91 (70), 77 (50).

Peak 3- 2D maps

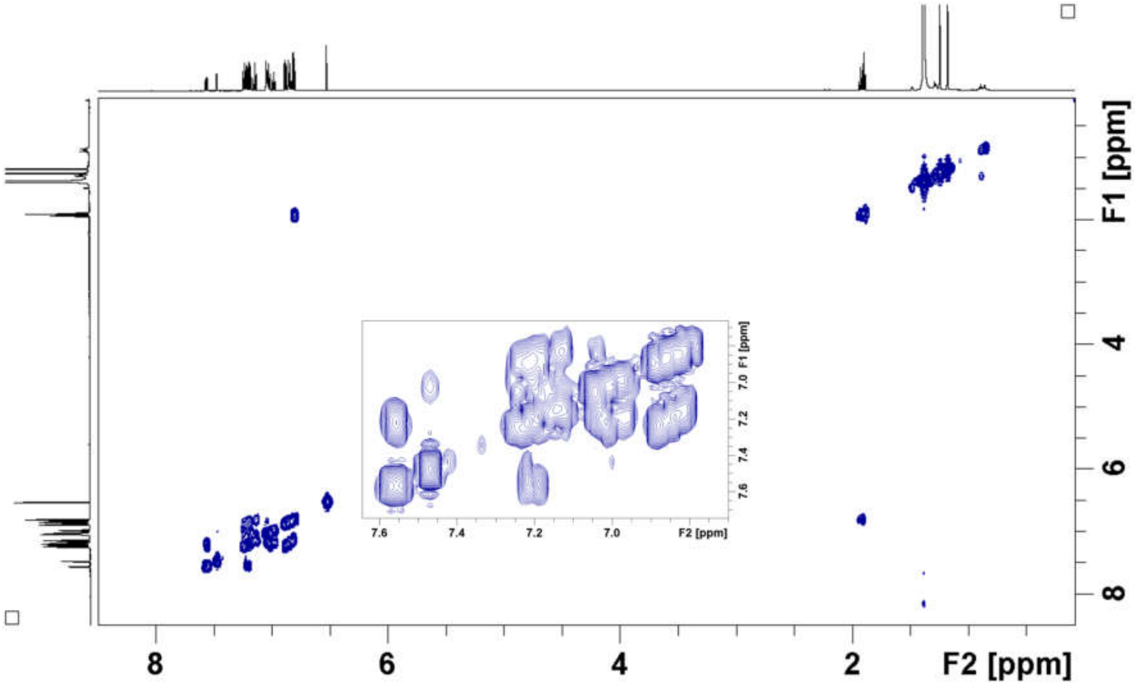

Figure S16- COSY (600 MHz, C_6D_{12}, 300 K) map of peak 3.

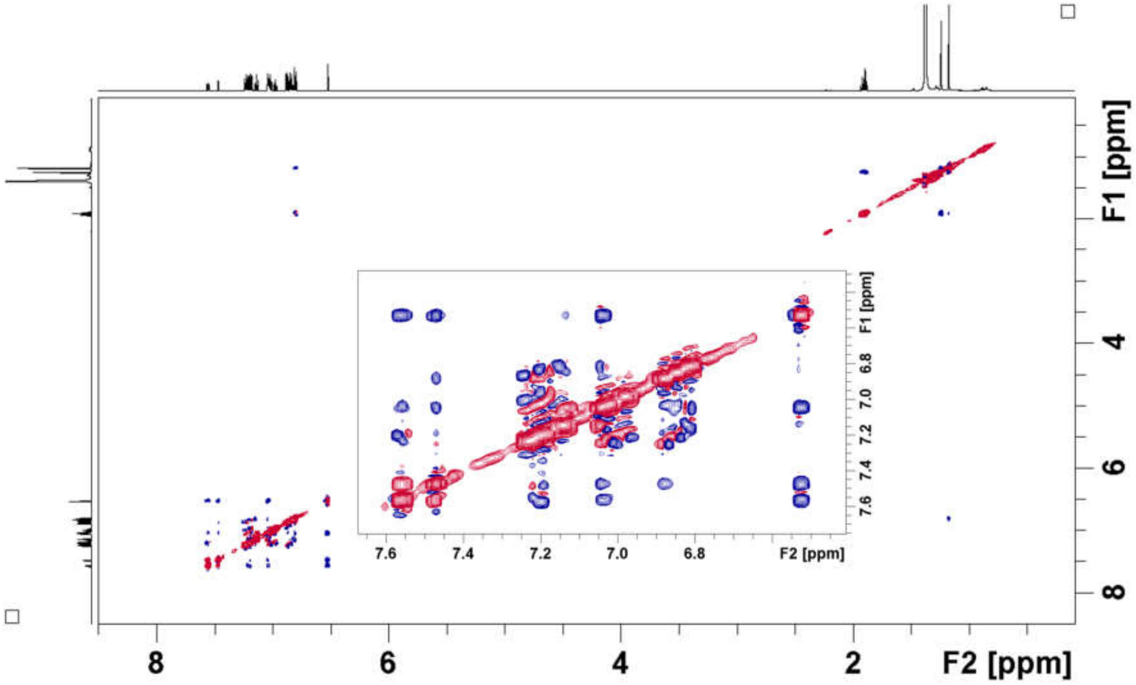

Figure S17- ROESY (600 MHz, C_6D_{12}, 300 K, mixing time = 300 ms) map of peak 3.

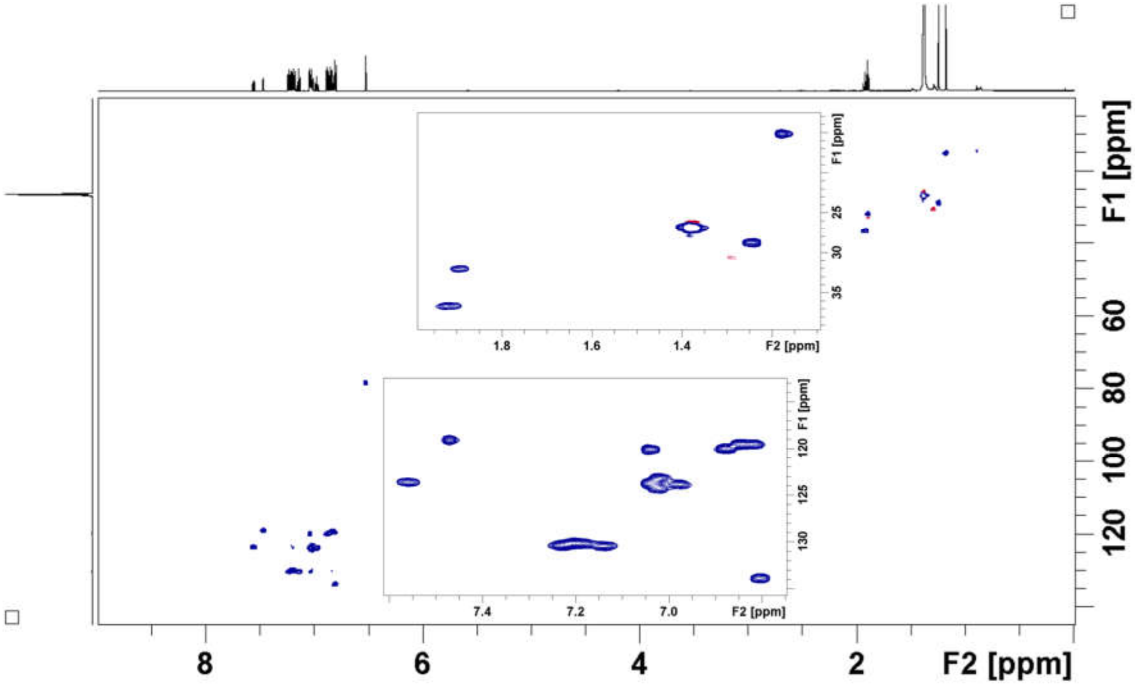

Figure S18- HSQC (600 MHz, C_6D_{12}, 300 K) map of peak 3.

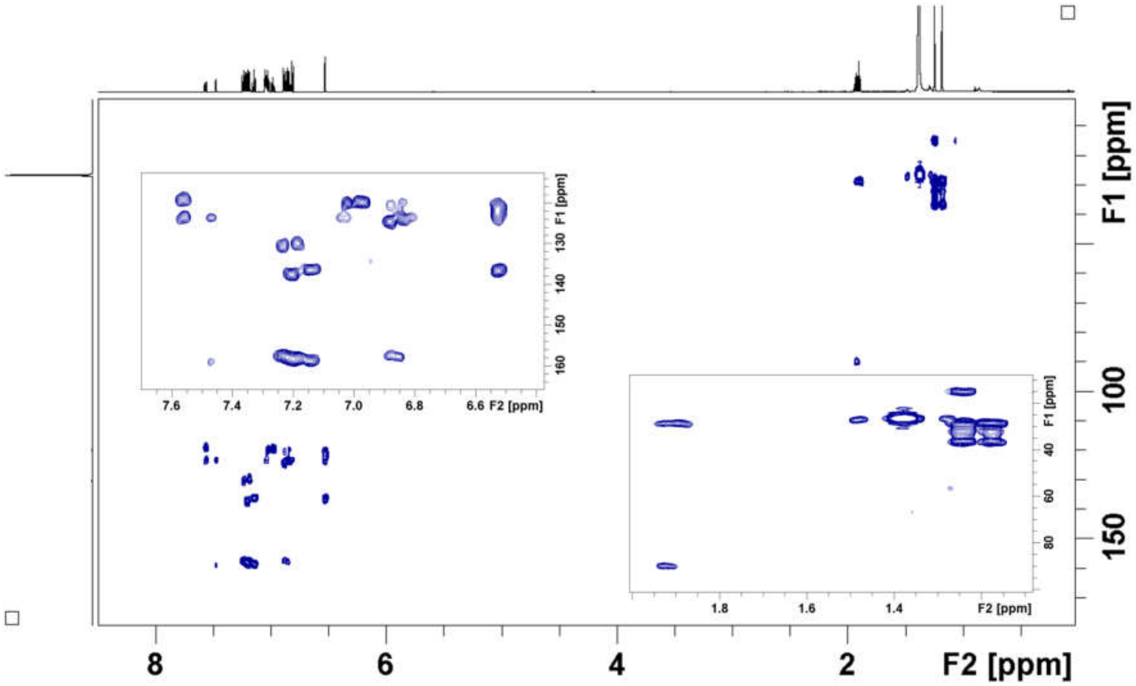

Figure S19- HMBC (600 MHz, C_6D_{12}, 300 K) map of peak 3.

Peak 5- 2D maps

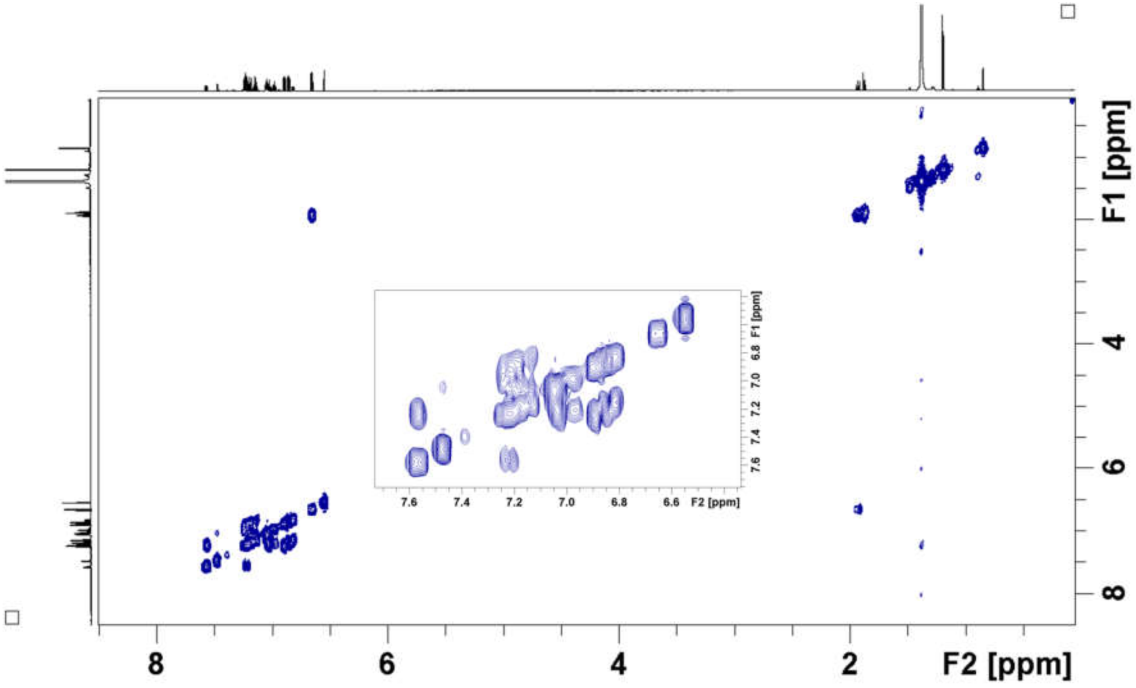

Figure S20- COSY (600 MHz, C_6D_{12}, 300 K) map of peak 5.

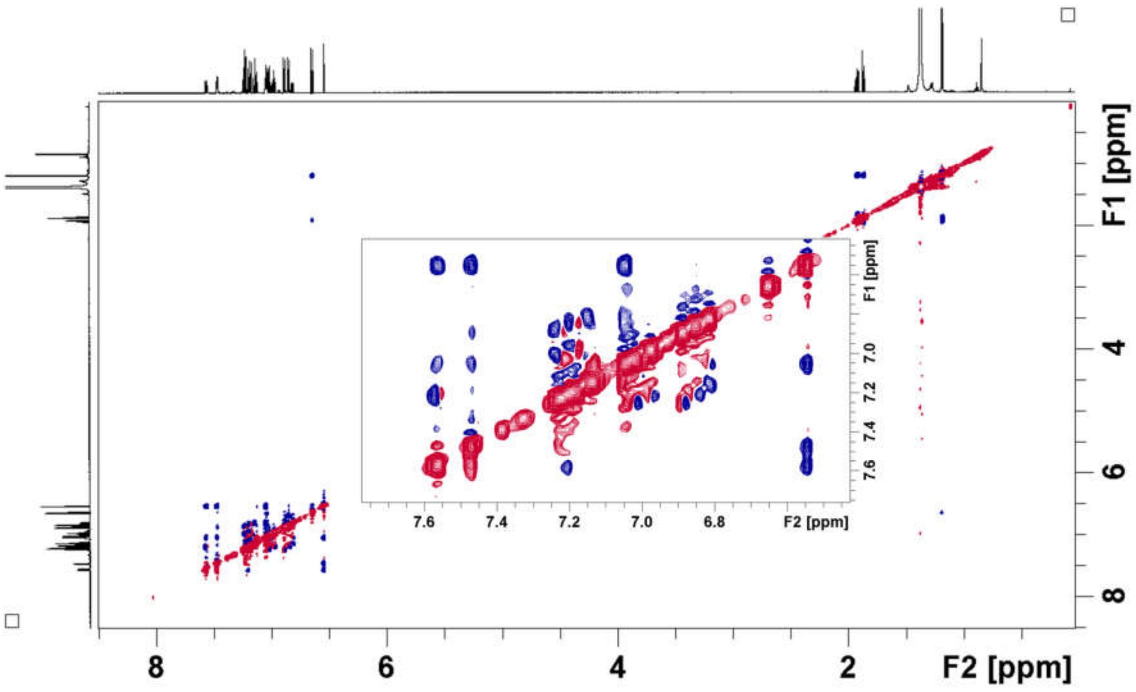

Figure S21- ROESY (600 MHz, C_6D_{12}, 300 K, mixing time = 300 ms) map of peak 5.

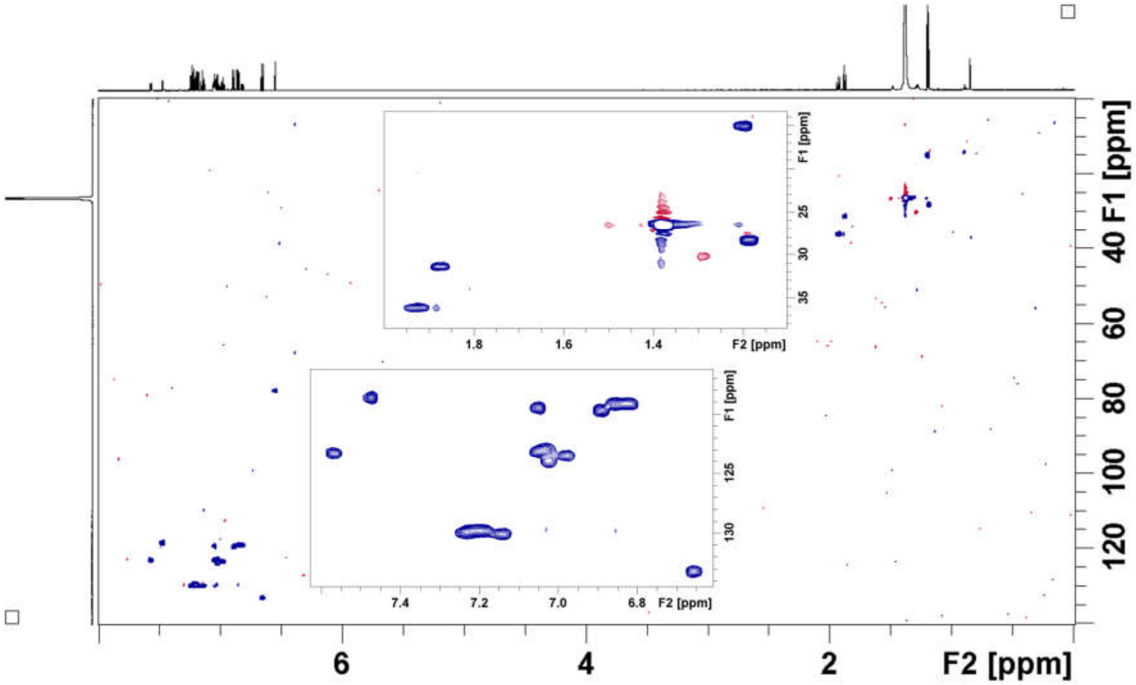

Figure S23- HSQC (600 MHz, C_6D_{12}, 300 K) map of peak 5.

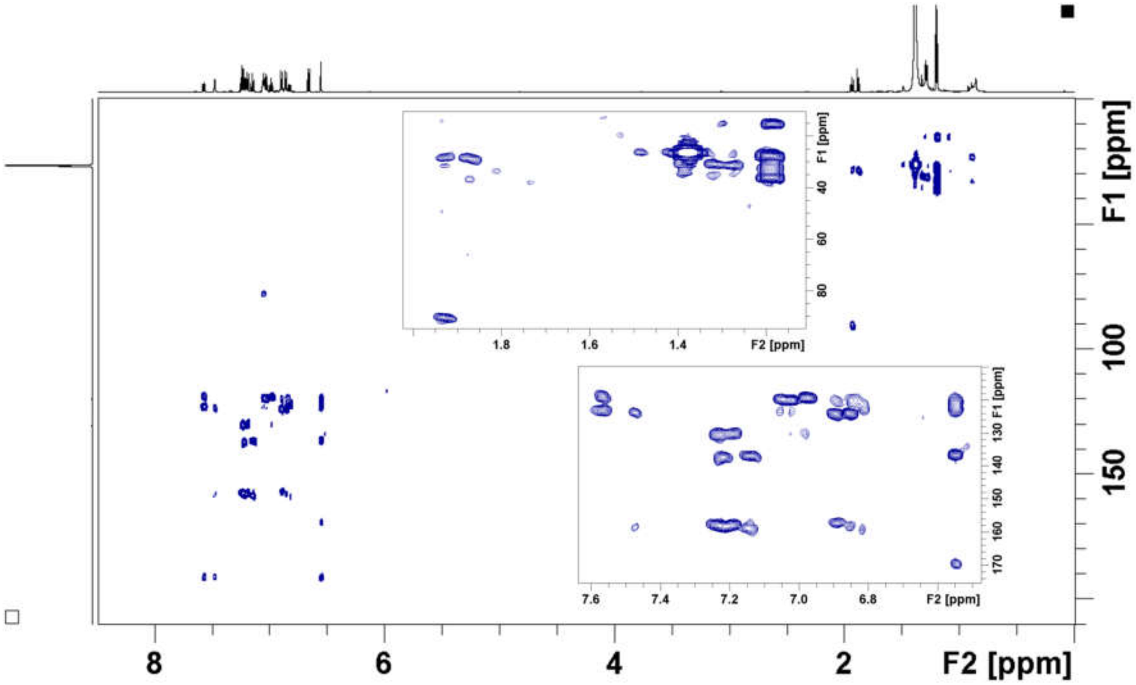

Figure S22- HMBC (600 MHz, C_6D_{12}, 300 K) map of peak 5.
